# Osthole Promotes Endochondral Ossification and Accelerates Fracture Healing in Mice

**DOI:** 10.1007/s00223-016-0189-4

**Published:** 2016-08-18

**Authors:** Zhongrong Zhang, Wing Nang Leung, Gang Li, Yau Ming Lai, Chun Wai Chan

**Affiliations:** 1 School of Chinese Medicine, Faculty of Medicine, The Chinese University of Hong Kong, Shatin, Hong Kong, China; 2 Department of Orthopaedics and Traumatology, Faculty of Medicine, The Chinese University of Hong Kong, Shatin, Hong Kong, China; 3 Department of Health Technology and Informatics, The Hong Kong Polytechnic University, Hung Hom, Hong Kong China

**Keywords:** Osthole, Fracture healing, Endochondral ossification, Bone mineral density, Molecular imaging

## Abstract

Osthole has been found to restore bone mass in preclinical osteoporotic models. In the present study, we investigated the effects of osthole on bone fracture repair in mice. Adult C57BL/6 mice were subjected to transverse femoral fractures and administrated orally with 20 mg/kg osthole and vehicle solvent daily from week 1 post-operation. Fracture callus were analyzed by plain radiography, micro-computed tomography, histology, molecular imaging and immunohistochemistry and tartrate-resistant acid phosphatase staining. Results demonstrated that osthole treatment enhanced removal of cartilage and bony union during reparative stage without significant interfering on remodeling process. In vivo molecular imaging showed bone formation rate of the treatment group was almost twofold of control group at week 2 post-operation. Osthole augmented the expression of alkaline phosphatase and collagen type X in hypertrophic chondrocytes as well as expression of bone morphogenetic protein-2, osteocalcin and alkaline phosphatase in osteoblastic cells, indicating it promoted mineralization of hypertrophic cartilage and woven bone growth simultaneously during endochondral healing. In summary, osthole promotes endochondral ossification via upregulation of maturation osteogenic marker genes in chondrocytes and subsequently accelerates fracture repair and bony fusion.

## Introduction

Bone regeneration takes place in consequence of osteoporotic fragility, trauma or orthopedic surgery [1–3]. Osteoporotic facture in elder people can lead to further disability and early mortality. It has become a significant global health problem since prevalence of osteoporosis is estimated over 200 million people worldwide and is continuously growing with aging of population [4, 5]. These fractures can be associated with morbidity like impaired healing, delayed union or non-union, leading to extra pain, prolonged hospital stay and convalescence period.

Drugs suppress osteoclastic bone resorption especially bisphosphonates (BPs) are widely used in current standard treatment of osteoporosis [6]. However, the efficacy of anti-resorptive agents on growth and recovery of bone mineral density (BMD) is limited, which is considered less than 2 % every year [7]. Besides, long-term treatment of these agents was reported not only reduce osteoclasts number but also significantly reduce osteoblasts number and subsequent bone formation [8]. Moreover, further clinical concern arises when fragility fracture occurred in osteoporotic patients who usually have poorer healing ability. Accumulating clinical reports suggested that higher risks of atypical femur fractures and jaw osteonecrosis were associated with prolonged BP treatment [9, 10]. Numerous experiments on animal models also indicated BP administration delay callus remodeling and may have negative effect on bone healing process [11–13]. Therefore, new therapeutic agents that can inhibit bone resorption as well as facilitate bone repair are needed, which are more desirable in treatment of fragility fracture caused by osteoporosis.

Medicinal plants have been suggested as large source of potential agents against osteoporosis [14]. Osthole is a naturally derived coumarin, which is found medicinal plants such as *Cnidium monnieri*, *Angelica archangelica* and *Angelica pubescens*. Total coumarins extract from *C. monnieri* fruit was reported be effective for prevention of bone loss in both ovariectomy and glucocorticoids-induced osteoporosis rat models [15, 16]. It was then suggested that the anti-osteoporotic effect was attributed to the major bioactive coumarin osthole, and this chemical was as effective as 17β-estradiol in suppressing bone loss in ovariectomized (OVX) rats [17, 18]. The anti-osteoporotic effect of osthole was further confirmed by Tang et al. [19], and they also suggested that osthole had anabolic effect since local injection of osthole increased new bone formation in mouse calvaria. Additionally, studies in cell models indicated osthole-induced osteoblastic differentiation by upregulation of osteogenic genes such as osteocalcin (OCN), alkaline phosphatase (ALP) and collagen type I (Col-I) by activation of bone morphogenetic proteins (BMP) pathway [19–21]. These experimental findings all suggested that osthole is a potential anabolic agent applicable for both treating osteoporosis and enhancing bone formation. In the present study, we examined the effect of osthole on bone healing process using a mouse mid-shaft femoral fracture model and investigated the possible mechanism comparing to the previous in vitro studies.

## Materials and Methods

### Animal Model

C57BL/6 mice were obtained from the Laboratory Animal Services Center of the Chinese University of Hong Kong. The animal study was approved by Animal Experimentation Ethics Committee (12/002/DRG-5). All mice were first acclimatized and housed at the research animal laboratory during the experimental period. Open osteotomy at femur diaphysis modified from previous study [22] was performed on 12-week-old male C57BL/6 mice to establish a stable femoral open fracture model. Briefly, mouse weight of 25–30 g was general anaesthetized with intraperitoneal injection of ketamine (67 mg/kg body weight) and xylazine (13 mg/kg body weight). After shaving and sterilization of right leg, a lateral incision was made and a 25-gauge needle was inserted retrograde into the intramedullary canal from knee articular surface for internal fixation. Diaphysis of the femur was exposed and an air pen driven oscillating saw (Synthes Holding AG, Zuchwil, Switzerland) was used to create a transverse mid-shaft fracture under irrigation with sterile 0.9 % saline solution. Digital X-ray radiography was taken to confirm the alignment of the bone. Absorbable sutures were used to close the intramuscular septum and skin incisions.

### Drug Administration and Specimen Harvest

Osthole (UHPLC 98 %) used in animal administration was purchased from LKT Laboratories, Inc. (MN, USA). Osthole reagent was freshly prepared by dissolution with 0.5 % (v/v) Tween 80 (Sigma-Aldrich, MO, USA) in distilled water. Three dosages of osthole, 5 (low), 20 (middle) and 50 (high) mg/kg of body weight (derived from Li et al. [18]) were given to mice by daily oral gavage from week 1 (day 7) post-osteotomy until euthanasia, and mice in control group were fed with vehicle solvent only. Body weights were monitored throughout whole experimental process.

Animals in control and treatment groups were subject to X-ray radiography, micro-computed tomography (µ-CT), histology and molecular imaging assessments. Fractured and contralateral femurs were harvested at the end of experiment at week 2, 3 and 4 post-operation. Soft tissues were removed from the operated and contralateral femurs. The specimens were fixed within 4 % paraformaldehyde (Life Technologies, NY, USA) solution for 24 h. Internal fixation needle was removed from the intramedullary cavity and the femur specimens were stored in 70 % ethanol for µ-CT and histologic processing.

### Plain Radiography

**Fig. 1:**
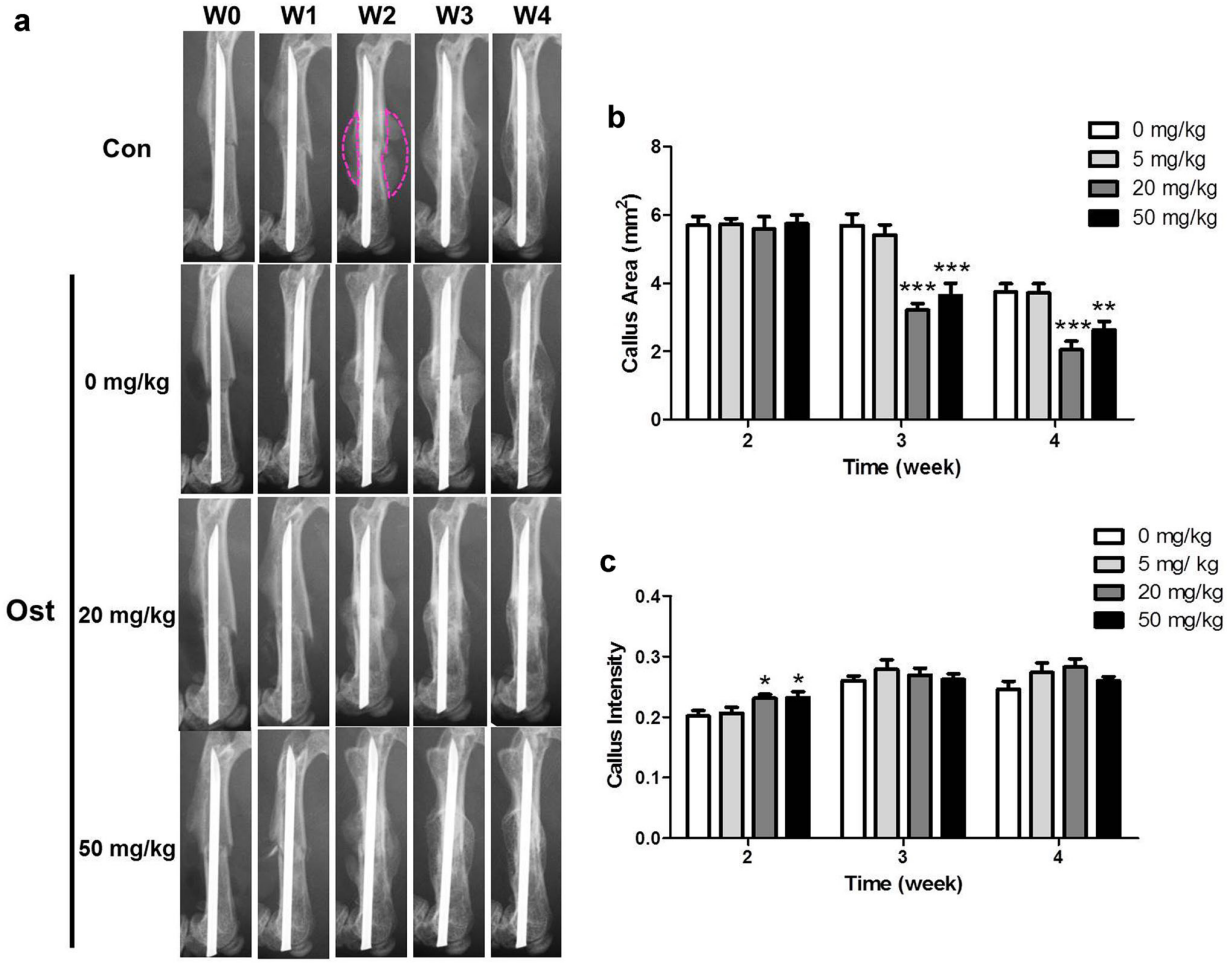
Osthole administration accelerated fracture healing process. Mice received femur osteotomy were oral administrated with 0 (control), 5, 20 and 50 mg/kg body weight of osthole from postoperative week 1. **a** Representative serial radiographs of one mouse from every group from week 0–4; *dotted line* callus ROI for analysis. **b**, **c** Area and pixel intensity of callus ROIs (mean ± SD, *n* = 10); two-way ANOVA followed by Tukey’s test, *p < 0.05, **p < 0.01, ***p < 0.001 compared to control group

Serial radiographs were conducted throughout the experiment period from osteotomy surgery (week 0) to euthanasia. Radiographs of animals from control, low, middle and high groups were taken weekly from week 0 to week 4 using the digital radiographic function of in vivo Multispectral FX PRO system (Carestream Health, NY, USA). The callus area and pixel intensity of the fractured femur was measured at each time point, by outlining the callus by region of interest (ROI) (shown with pink dotted line in Fig. 1a) with Carestream Molecular Imaging (MI) Software.

### In vivo and Ex vivo Molecular Imaging

In vivo bone growth were imaged and analyzed with two commercial fluorescent in vivo bisphosphonate imaging probe OsteoSense^®^ 680 Ex (OS680) and OstenSense^®^ 800 (OS800) (PerkinElmer, MA, USA) with two distinct wavelengths modified from suggested protocol and previous study [23]. Briefly, anesthetized mice were injected retro-orbitally with dissolved fluorescent probes (10 nmol/kg) 48 h before imaging. Hair was removed from the hind legs. Florescent signals and X-ray images were captured by combination of multispectral fluorescence and digital radiograph function of in vivo Multispectral FX PRO system. After anesthesia, signals of injected Os680 were detected in vivo with excitation wavelength at 650 nm and emission at 700 nm at week 2, while Os800 was injected and detected (excitation 760 nm/emission 830 nm) at week 3. At week 4, both florescent signal of Os650 and Os800 in both sides of femur were measured ex vivo after femurs were harvested. Florescent and radiographic images obtained were overlayed and analyzed with Carestream MI Software. ROIs of same area were selected from operated and contralateral femurs versus surrounding skin (as background signal). Os signal intensity of fractured right femurs was normalized by intensity of contralateral side.

### Micro-Computed Tomography (µCT) Analysis

Harvested and fixed bone specimens of week 2, 3 and 4 were subject to µCT scanning using MicroCT40 (Scanco Medical, Switzerland). The scan range covered a 6 mm thickness with the center at fracture line at an isotropic resolution of 10 µm. The contoured ROI were selected from 2D CT images, and 3D reconstruction were performed with a low-pass Gaussian filter (Sigma = 1.2; Support = 2). Low attenuation threshold of 130 and high attenuation threshold of 250 was set to distinguish newly form mineralized callus from old cortical bone referring to previous study [24]. Quantitative analysis was performed covering 250 slices above the fracture line and 250 slice below. Callus total volume (TV) identified the volume of newly formed tissues (callus) and low-density bone volume (BV_l_) identified the volume of newly formed mineralized tissue in callus. TV and BV_l_ were both calculated by reduction of the volume in fractured femur with the volume of the contralateral cortical bone. Tissue mineral density (TMD) measured the volumetric density of calcium hydroxyapatite (CaHA) in total tissue including both newly formed callus and old cortical bone; bone mineral density (BMD) measured volumetric density of CaHA in total mineralized tissue (bone). TMD and BMD were expressed as percentage of contralateral intact bone.

### Histology and Immunohistochemistry

Fixed bone specimens were decalcified with calcium chelating solution (0.5 M EDTA/NaOH, pH 7.5) for 2 week. Decalcified bones were then dehydrated and embedded in paraffin wax using Leica EG Embedding Center (Leica Microsystem, Wetzlar, Germany). Paraffin blocks were sectioned into 5 µm slices and mounted on glass slides. The sections were deparaffinized and triple stained with hematoxylin and eosin (H&E) along with alcian blue (Sigma) specific for cartilage tissue [25]. The histomorphometry analysis was performed by a blinded observer using Zen2012 (Zeiss, Oberkochen, Germany).

For immunohistochemistry (IHC), sections were deparaffinized and rehydrated in phosphate buffered saline (PBS). After endogenous peroxidases were quenched with 3 % H_2_O_2_/MeOH, antigen retrieval was performed according to the suggestions from primary antibody producer. After nonspecific binding blocked with UltraVision protein block (Thermo Scientific, MA, USA) or incubation solution for 30 min, sections were incubated overnight at 4 °C with diluted solution of primary antibody against BMP-2, ALP, OCN, Col-I, Col-X Cathepsin K (CTSK) (Abcam, Cambridge, UK) and solution without antibody as negative control. The sections were then incubated with horseradish peroxidase conjugated secondary antibody (Santa Cruz biotechnology, CA, USA) for 30 min at room temperature. IHC signal was developed with Liquid DAB + Substrate Chromogen System (Dako, CA, USA), counter-stained with hematoxylin and subjected to blinded evaluation.

### Tartrate-Resistant Acid Phosphatase (TARP) Staining

Deparaffinized sections were stained using Sigma Acid Phosphatase, Leukocyte (TRAP) Kit following the instructions provided. Briefly, slide was firstly incubated in Solution A (125 µg/ml naphthol AS-Bl phosphoric acid, tartrate/acetate buffer) at 37 °C protected from light for 45 min and then subsequently incubated in Solution B (70 µg/ml diazotized Fast Garnet GBC base in tartrate/acetate buffer) for 5 min to develop the color. After counterstaining with hematoxylin, sections were mounted by aqueous mounting medium and evaluated microscopically within 1 day.

### Statistical Analysis

Statistical analysis was conducted using GraphPad Prism 5.0 (GraphPad Software, CA, USA) software and Excel (Microsoft, CA, USA). All data were obtained from 6 to 10 individual mice in control or treatment groups. Mean and standard deviation values (mean ± SD) were calculated for all statistically analyzed parameters. The differences between groups were analyzed using ANOVA with Turkey’s post hoc test or unpaired Student’s *t* tests. The *p* value less than 0.05 was considered statistically significant.

## Results

### Osthole Administration Accelerated Fracture Healing Process

To determine the effect of osthole gavage dosage on mice fracture repair, mice were treated with vehicle solvent and three dosage of osthole. Mice body weight and fractured bone morphology from 1 to 4 weeks were monitored and analyzed. Body weight in each group did not significantly change throughout experimental period. Serial radiographs of control and low-dose group demonstrated very similar morphology changing pattern (Fig. 1a). Clear callus contour could be defined at week 2 and at week 3 the callus became more radiopaque with similar size as compared with that of week 2. At week 4, callus size was found obviously reduced. Whereas in group treated with middle and high dose, the callus size at week 3 was apparently reduced as compared with week 2, which was much smaller than that in control group; and the callus size was further reduced at week 4 (Fig. 1a). The area and pixel intensity of callus ROIs was quantified and used to estimate the volume of callus and the quantity and density of bone in it (Fig. 1b, c). Callus area in middle and high group was significantly smaller than that of control group (5.69 ± 1.09 mm^2^) by 43.65 and 36.17 % of control, respectively. The intensity of callus ROIs at week 2 in middle and high group (0.2310 ± 0.0204 and 0.2320 ± 0.0311, respectively) was slightly higher than average control value (0.2016 ± 0.0228) with a statistical significance. Since middle and high dose of osthole did not have significant difference, middle dose (20 mg/kg/day) was selected as the optimum dose and applied in further assessments.

### Osthole Increased Bone Mineral Density and Promoted Bony Callus Formation

**Fig. 2:**
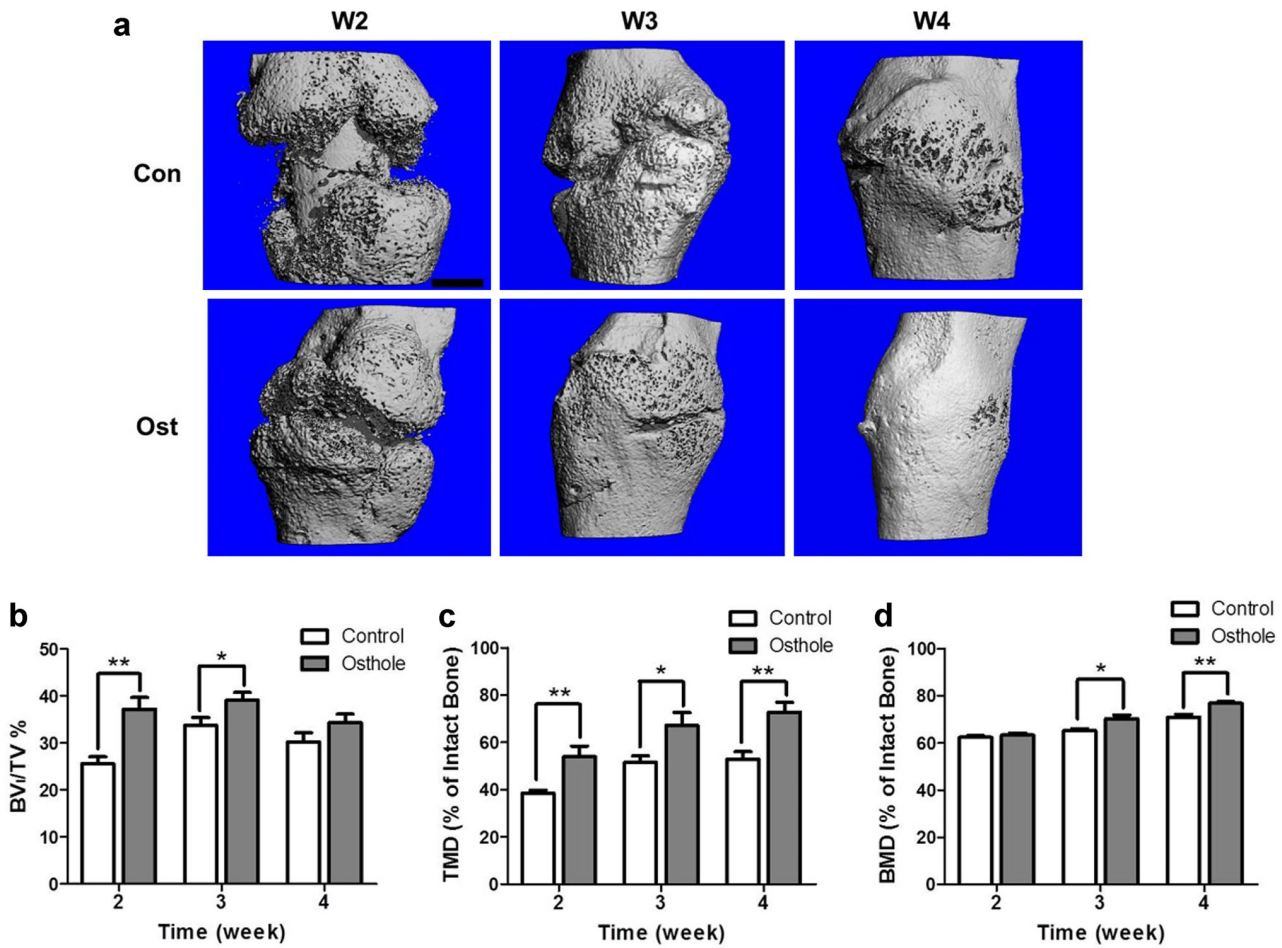
Osthole promoted callus bridging and increased bone density. Fracture calluses from control and osthole-treated mice were harvested at week 2, 3 and 4 post-osteotomy. **a** Representative 3D images of fractured femur from both groups at week 2, 3 and 4 (*Bar* 1 mm). **b**–**d** BV_l_/TV, TMD, BMD (mean ± SD, *n* = 6); one-way ANOVA followed by Tukey’s test, *p < 0.05, **p < 0.01

Reconstruction of µCT scan slices allowed a visual evaluation and comparison of fracture callus in control and osthole-treated mice. In the representative 3D image of control group, there was large gap between bony bridge in callus at week 2; the newly formed bone had not fully bridged until week 3, whereas higher proportion of mineralized bone in callus was observed in osthole group at week 2. Fractured gap had already completely surrounded by hard callus at week 3, and at week 4, the fractured bone was further remodeled and restored to original shape and texture (Fig. 2a). Analytical results of 3D radiography were generally consistent with the results from plain radiography. Average BV_l_/TV of control group reached peak value at week 3 and slightly decreased at week 4, similar to the trend of callus intensity obtained from analysis of 2D radiographic images; BV_l_/TV of osthole was significantly higher at week 2 and 3 by 45.27 and 15.87 %, respectively (Fig. 2b). TMD and BMD showed a steady increase along time in both groups, while comparing between groups demonstrated that osthole administration markedly raised TMD of fractured femur at each time point (Fig. 2c); BMD of treated mice was significantly higher than control at week 3 and 4 (Fig. 2d).

**Fig. 3:**
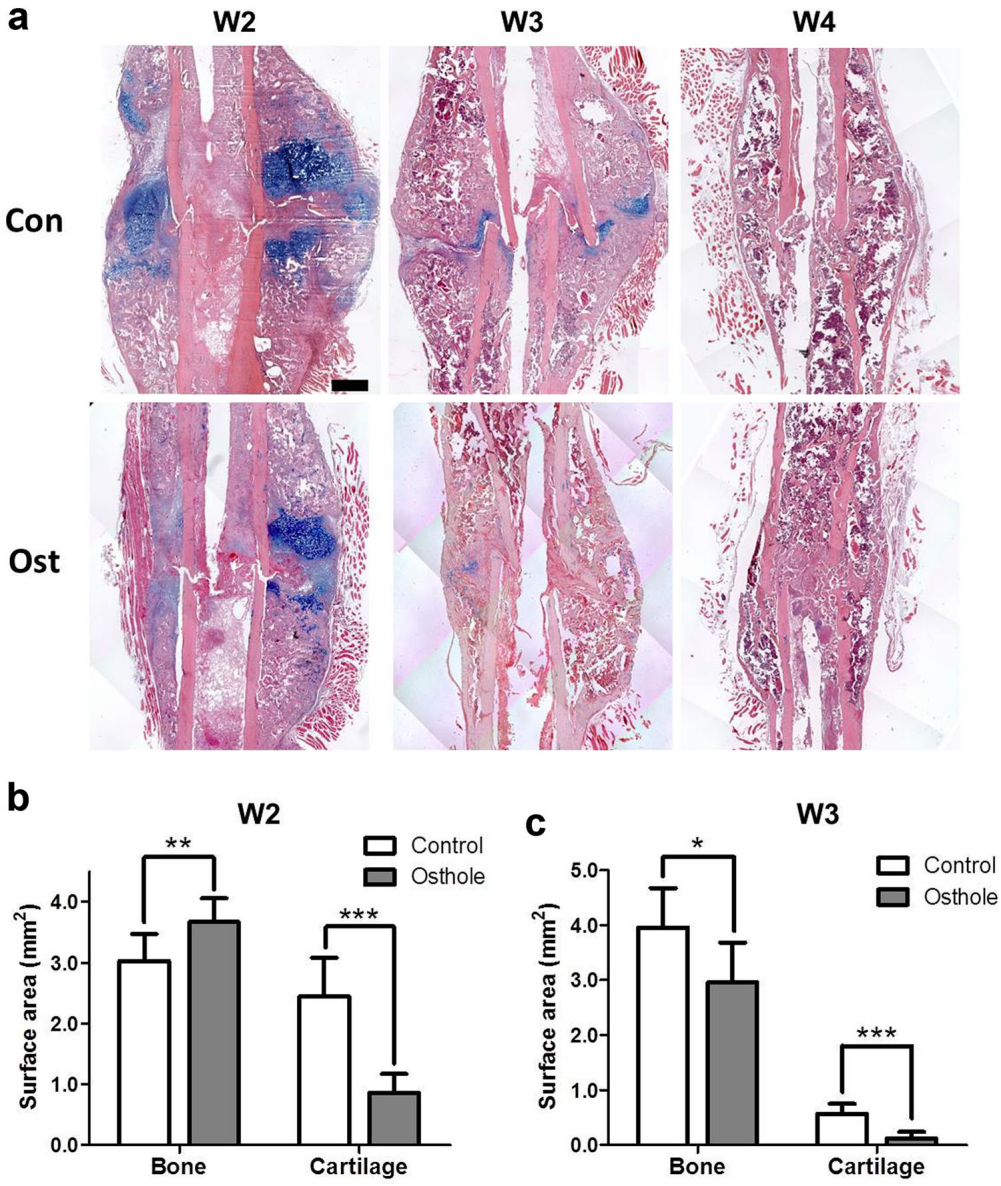
Osthole enhanced cartilage removal in reparative phase. Fracture calluses were harvested and decalcified at week 2, 3 and 4 post-osteotomy. Callus sections were tripled stained with alcian *blue* (cartilage) and H&E. **a** Representative images showed cartilaginous (*blue*) and mineralized (*pink*) callus from both groups at week 2, 3 and 4 (*Bar* 500 µm). **b**, **c** Surface area of cartilage and woven bone in callus at week 2 and 3 (mean ± SD, *n* = 6 or 8); unpaired Student *t* test, *p < 0.05, **p < 0.01, ***p < 0.001 (Color figure online)

Since radiographic evaluations were not able to identify cartilage and other soft tissues, histological sections of fractured bone were labeled with both cartilage and bone and histomorphometric analyses were performed. Histology results complemented µCT images: at week 2 post-fracture, control calluses contained similar amount of cartilaginous and bony tissues; at week 3 cartilages persisted in calluses; and by week 4 cartilages had diminished and callus size reduced indicating remodeling. Whereas osthole group exhibited obviously smaller total surface area of cartilaginous tissues at week 2, the callus had fully bridged with woven bone by week 3; bony callus was further remodeled and the cortical fracture gap was united (Fig. 3a). Histomorphometric analysis showed the bony callus area in osthole group was significantly larger than control group, while the cartilaginous callus area was much smaller at week 2 post-operation (Fig. 3b); the woven bone surface area in treatment group became smaller than control and the cartilaginous tissue almost diminished from callus at week 3 (Fig. 3c).

### Osthole Enhanced Osteoblastic Bone Formation and Promoted Endochondral Ossification

**Fig. 4:**
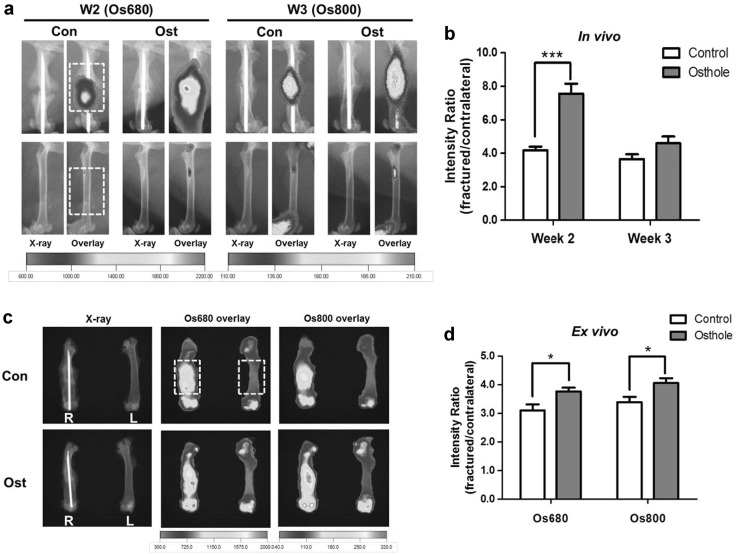
Osthole increased mineralization rate of callus. Mice in control and osthole group were both injected with Os680 and Os800 and subjected to in vivo imaging at week 2 and 3, respectively, and femurs were harvested after euthanasia at week 4 and subject to ex vivo imaging for both probes. **a**, **c** Representative overlay images of X-ray and florescent signals of both groups taken in vivo (**a**) and ex vivo (**c**), dotted line: the ROI for intensity quantification. **b**, **d** Intensity ratio (fractured/contralateral) of Os680 and Os800 (mean ± SD, *n* = 6); unpaired Student *t* test, *p < 0.05, ***p < 0.001

Osteosense (Os) was used to localize neo bone growth and to quantify growth rate in living animals. Due to long half-time of Os probes in bone and their narrow excitation and emission wavelength range, mice with osteotomy were dual-labeled with Os680 and Os800 at week 2 and 3, respectively. Positions of new bone formation were mapped in living animals by florescent signals, and bone growth rate was quantified by signal intensity ratio and compared between control and osthole group (Fig. 4a). The bone formation rate at week 2 in treatment group was markedly higher than that in control group by 80.72 %, which dropped at week 3 but still higher than control value by 25.95 % (Fig. 4b). Florescent signals of both probes were captured ex vivo after euthanasia at week 4 to eliminate the possible interference of soft tissues. The ex vivo florescence pattern of two probes more accurately demonstrated the new bone formation inside calluses. Os680 signals distributed around two ends of the callus, while Os800 signals located at the center of the callus near fracture line (Fig. 4c). Average values of fractured/contralateral fluorescent intensity ratio were obtained from both lateral and central views. Fluorescent intensity ratio of both Os680 and Os800 were relatively lower compared to their in vivo values, whereas for both probe the ex vivo intensity ratios of osthole group were significantly higher than those of control group (Fig. 4d).

**Fig. 5:**
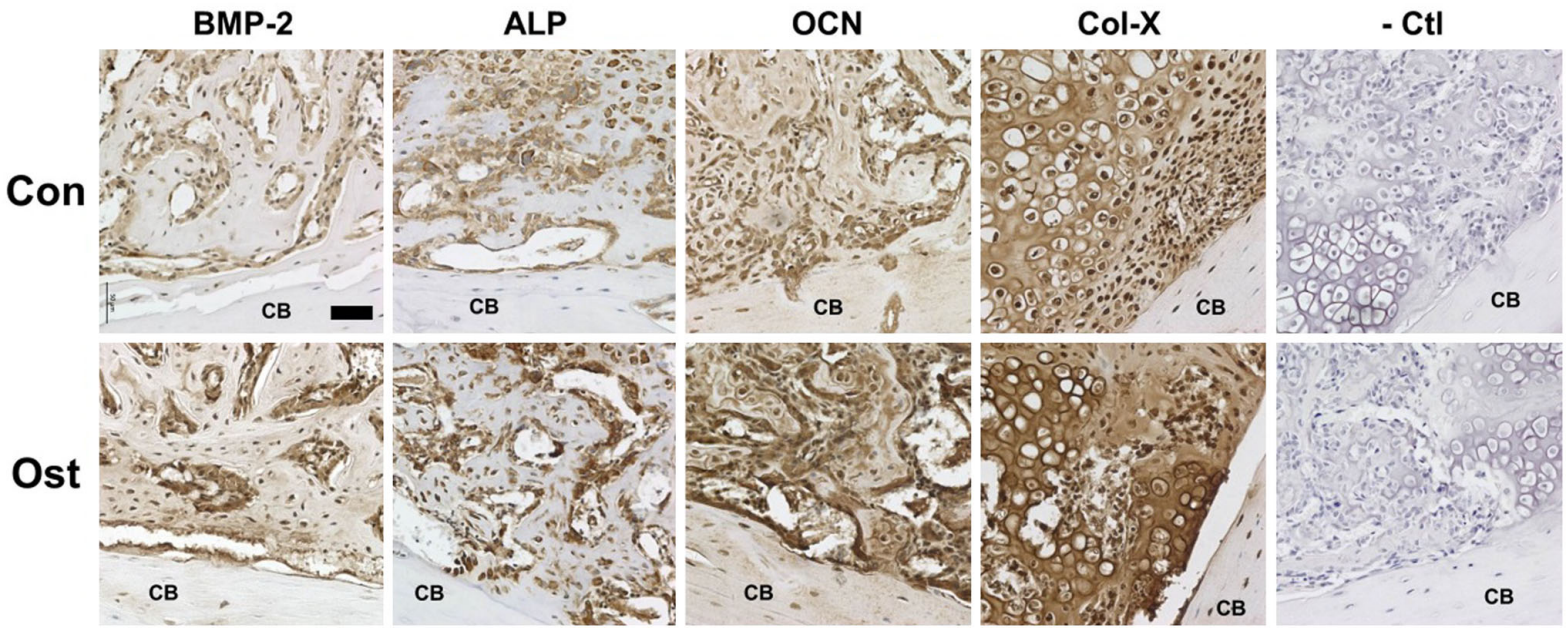
Osthole upregulated osteogenic and chondrogenic markers during endochondral bone formation. Fracture calluses were harvested and decalcified at week 2 post-osteotomy. Callus sections were blotted with BMP-2, ALP, OCN, Col-X antibody and negative control and then counterstained with hematoxylin. Representative images showed both cortical bone (CB) and newly formed cartilage/bone tissues (*n* = 4–6; *Bar* 50 µm; *CB* cortical bone)

Immunohistochemistry (IHC) of osteogenic marker were performed and compared between control and treatment group at week 2. As shown in Fig. 5, BMP-2 mainly expressed in osteogenic cells surrounding woven bones; ALP specifically expressed around hypertrophic cartilaginous tissues and newly formed bone tissues; OCN expressed throughout the whole callus which is highly expressed in osteogenic cells but also found in bone matrix. In osthole-treated group, expression of BMP-2, ALP and OCN was all observably higher than those in control group. Osthole treatment also evidently increased OCN expression in newly formed bone matrix but not affected cortical bone. Col-X and Col-I were also accessed by IHC at week 2. Col-X was only highly expressed in hypertrophic chondrocytes and cartilaginous matrix, and Col-I expression were found mainly in soft tissues but also hard tissues in callus. Interestingly, osthole administration dramatically promoted the expression of Col-X at week 2 but have no marked effect on Col-I expression (data not shown) (Fig. 5).

### Osthole Had No Effect on Osteoclast Number and Did Not Delay Callus Remodeling

**Fig. 6:**
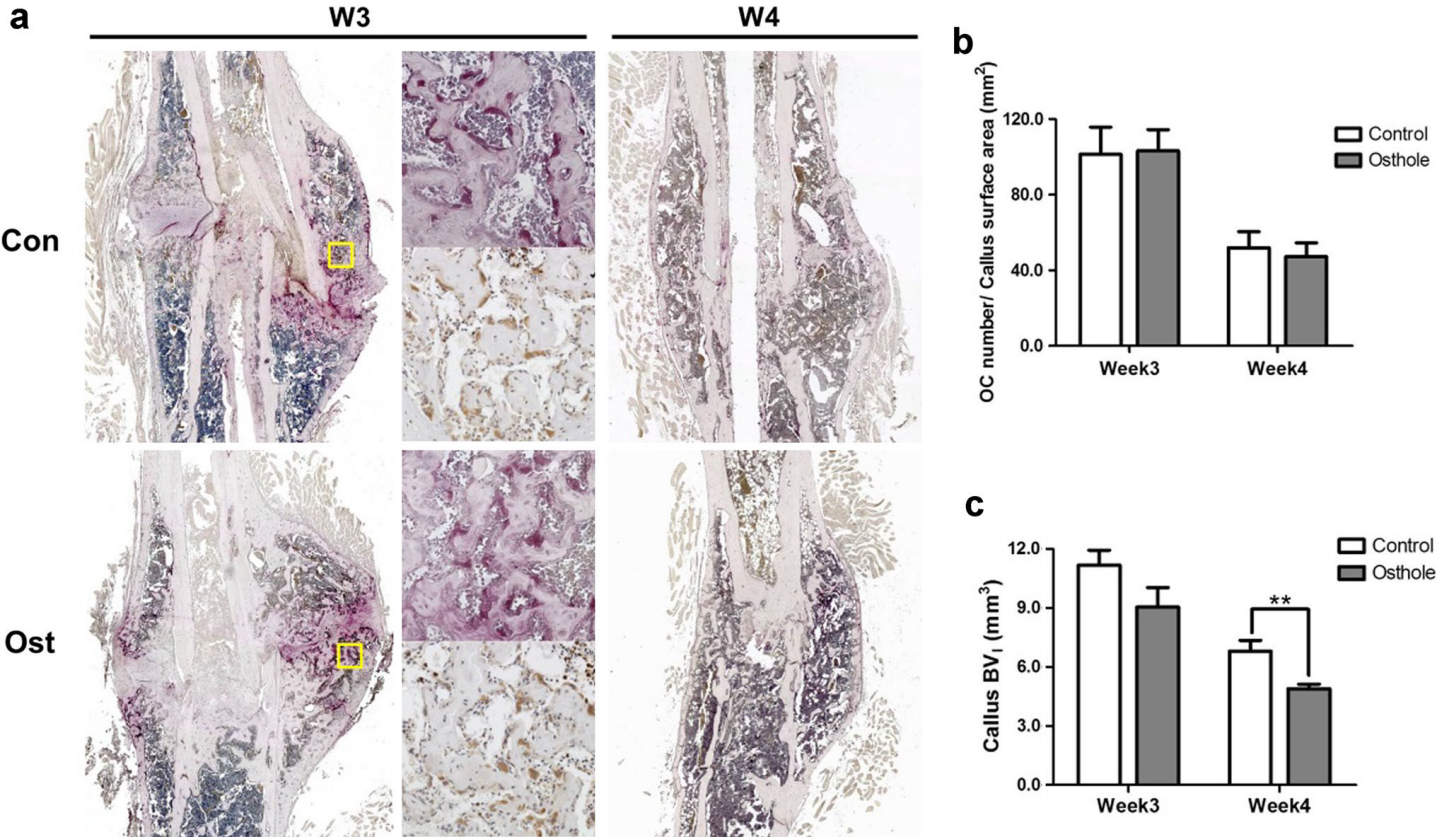
Osthole did not affect osteoclasts abundance and remodeling progress in callus. Fracture calluses from control and osthole-treated mice were harvested at week 3 and 4 post-osteotomy. **a** Representative TRAP staining and immunohistochemical staining of CTSK; small rectangular indicated area enlarged, CTSK immunostainings were taken at close position from the sample specimen (*n* = 4, *Bar* 50 µm) **b** TRAP positive osteoclasts number per mm^2^ fracture callus surface area in control and osthole group (mean ± SD, *n* = 4). **c** Quantification of low-density bone volume at week 3 and 4 (mean ± SD, *n* = 6); unpaired Student *t* test, **p < 0.01

To examine the influence of osthole administration on osteoclastogenesis during fracture callus remodeling, TRAP staining of callus histological sections at week 3 and week 4 was performed and IHC staining of osteoclastic marker CTSK was conducted to further confirm the results. Figure 6a showed that osteoclasts distribution of two groups was similar at both time points. At week 3, TRAP positive osteoclasts located near fracture site with high density, at the surface of newly formed woven bone. By week 4, osteoclasts abundance markedly dropped and osteoclasts distributed relative evenly in the whole callus. CTSK immunostaining demonstrated similar osteoclasts number and distribution. Quantification of the osteoclasts number expressed as cell number per unit callus surface area (mm^2^). Osteoclasts number was 101 ± 14 (Con) compared to 103 ± 11 (Ost) at week 3; and 52 ± 9 (Con) compared to 47 ± 7 (Ost) at week 4, which did not show significant difference between control and treatment group (Fig. 6b).

Plain X-ray and µ-CT imaging of bony callus in osthole group both demonstrated reduction of callus size from week 3 to week 4, suggesting callus remodeling was not delayed after osthole gavage (Figs. 1a, b, 2a). Low-density newly formed bone volume was 11.18 ± 1.85 (week 3) and 6.80 ± 1.35 (week 4) mm^3^ in control group; and 9.04 ± 2.43 and 4.89 ± 0.57 mm^3^ in osthole group, the volume in osthole group was significantly lower than that in control group at week 4 (Fig. 6c). The average reduction volume of woven bone in control and osthole group was 4.38 and 4.15 mm^3^, respectively, which was close to each other, indicating the remodeling efficacy was not significant affected.

## Discussion

Osteoporotic fractures are among the most prevalent health conditions in elder population. Although BPs are used with satisfactory results for restoring bone density in osteoporosis therapy, their application on patients with fragility fractures is contentious having been reported to cause compilations during bone repair and to delay healing progress. Therefore, anabolic agents that stimulate bone formation, such as BMP-2,-7 and parathyroid hormone (PTH), are suggested enhancing bone healing process, reducing fracture-associated complications and hence benefiting normal and osteoporotic fracture repair when applied alone or combined with BPs [26]. However, in spite of the potency of these anabolic growth factors, they have deficiencies like drug delivery limitation, safety concerns and cost-efficient issues. Safer anabolic agents with substantial cost and easy way of administration are thereby still required. Osthole has been shown to stimulate local bone formation [19] and inhibit osteoporotic bone loss [18, 19] as well, which makes it an ideal potential candidate for osteoporotic fractures treatment. We hypothesized osthole is an osteoanabolic agents that promote endochondral bone growth in fracture repair.

Given the fact that osthole injection accelerated osteoid formation and mineralization in mouse calvaria surface [19], we hypothesized that oral gavage of osthole enhance osteoblastic bone formation in fracture healing. Endochondral fracture repair is nevertheless a complex process that involves a serial of biological events requiring interaction of different tissue and cellular systems other than osteogenic cell lineages. Hence, osthole was given from week 1 post-fracture to reduce the effect of osthole on early events in inflammatory phase. Healing progress was evaluated from 1 to 4 weeks covering the whole reparative phase and the beginning of the prolonged remodeling phase. Three different dosages in this study were adapted from oral application of osthole on rat osteoporotic model [18]. Area and pixel intensity of ROIs were quantified for estimating the volume and average mineral density of callus accordingly [27], to study the dose-dependent effect relationship of osthole on fracture repair. Results showed higher mineral density and smaller volume of callus in middle and high dose, whereas the middle and high group did not show significantly difference. This is probably because of the low oral bioavailability of osthole which limit the actual absorption [28, 29]. Middle dose was selected for the following assessments in this study.

Our radiographic and histomorphometric analyses at week 2 and 3 demonstrated a comprehensive evaluation of fracture healing process that showed clear acceleration of bony callus bridging and removal of fibrocartilage/cartilage by osthole treatment during reparative progress. It is worth mention that osthole not only increased the proportion of bone content in callus at week 2 and 3, but also significantly raised the average BMD of newly formed bone tissues. This result agrees with the previous reports that oral intake of osthole improved BMD and biomechanical properties in osteoporotic animal model [18, 19]. Near-infrared florescence labeled pamidronate probes (Os) allow to in vivo localize new bone formation and measure ossification rate in living animal. It has also been well established that bisphosphonates deposit into areas of new bone formation [30]; also near-infrared florescence molecules have been widely used in in vivo imaging for good photon propagation through living tissue and high signal to background ratio [31]. Furthermore, due to narrow excitation/emission wavelength range of Os probes and their long half-time of in bone, fractured femurs were dual-labeled and examined with Os680 and Os800 at week 2 and 3, respectively; and ex vivo signals of both probes were taken after bones harvested from animals. Average ossification rate in callus treated with osthole at week 2 was almost twice as much as control, which was close to the osthole-induced increasing of mineral appositional rate obtained by calcein labeling [19]. Mineralization rate of osthole group dropped remarkably at week 3, indicating end of reparative phase and transition to remodeling phase. It was still higher than control group in average by 25.95 %, but statistical significance was not observed because of large ingroup variations. Intensity ratio calculated from ex vivo signals were much lower compared to in vivo values for both probes in both group. It was probability because florescence molecules faded with time and some probes had dissolved back to surround fluid due to bone remodeling. Nevertheless, osthole-treated callus still showed significant higher ex vivo ratio than control ones.

Expressions of osteogenic-related proteins were examined with IHC to determine the mechanism by which osthole accelerate fracture repair. At week 2 post-osteotomy, both cartilage and woven bone existed in calluses from both control and treatment group. In hypertrophic chondrocytes and matrix, both Col-X and ALP were much higher expressed in osthole group than control group. Both of them are terminal differentiation markers for hypertrophic chondrocytes [32–34], indicating more rapid calcification in cartilage matrix. ALP also higher expressed in osteoblastic cells surrounding new bone islands, along with growth factor BMP-2 and another osteogenic marker OCN, all suggested more active woven bone growth rate in new bone area. Upregulation of BMP-2, ALP and OCN in osteoblasts inside callus echoed with the results obtained from in vitro osteoblastic cell models, while on the other hand, there was no observable difference in expression of Col-I between osthole and control group, which was not consistent with the findings on primary osteoblasts and osteoblast-like cell lines [19–21]. Col-I is the main collagen type in bone developing cross-linkage, and the effect of osthole on total Col-I content was probably not significant enough, even if osthole administration did invoke the expression in chondrocytes and osteoblasts. Hypertrophic chondrocyte formation in soft callus is an essential process of endochondral ossification in fracture healing. Hypertrophy of chondrocyte can be mediated by β-catenin/Runx2/smad pathway with downstream expression of Col-X [35]. Osthole has been reported to activate β-catenin, Runx2, smad1/smad5 in osteoblastic cells [19, 21]. Additionally, osthole-induced BMP-2 upregulation might also facilitate in early stage of chondrocyte hypertrophy [36]. Together, IHC results indicated that during reparative phase osthole enhances mineralization of hypertrophic cartilage as well as woven bone formation by augmenting expression of Col-X (chondrocyte maturation marker) and osteogenic markers.

Our study also demonstrates that osthole has no significant effect on formation and activity of osteoclasts during remodeling stage. Osthole gavage maintained the number of osteoclasts and osteoclastic bone resorption from week 3 to week 4. In addition, digital radiography shows advanced remodeled callus in both size and texture at week 4 in treatment group. This result is unexpected because inhibitory effect of osthole on osteoclastogenesis was reported in both osteoclast cell culture and osteoporotic rodent [19, 37]. Considering osteoclastogenesis is highly modulated by osteoblastic cells and several osteoblast secreted proteins bind to osteoclast surface receptor directly enhancing bone resorption [38, 39], it is likely that osthole-activated osteoblastic activity may also stimulate osteoclastic activity that overcome inhibitory effect of osthole on osteoclastogenesis. Although previous studies suggest that osthole exhibits BP-like osteoclastic inhibitory effect, the histological changes of osthole-treated callus is totally different from alendronate-treated fracture healing. Alendronate maintains callus size and increase mineral density [11, 40], but does not provide stimulation effect on osteoblasts.

On the other hand, considering the effects of osthole on BMD and strength of untraumatized cortical and cancellous bone and more dramatic promotive activity on bone healing and union, the action of osthole on bone is similar to the anabolic activities of PTHs [41–43]. Furthermore, both of their mechanisms on osteoblast differentiation are suggested closely associated with β-catenin signaling [44–46]. Consequently, osthole may share the similar potential as PTH on preventing fragility fracture and enhancing healing of fractures when normal process fails in patients with osteoporosis [43] and can be applied in prolonged therapy as BPs to restore BMD. A limitation of this study is it mainly focused on endochondral ossification stage, the effects of osthole on early events in fracture healing including angiogenesis and chondrogenesis should also be evaluated in detail to realize complete mechanism of application of osthole on fracture healing.

In conclusion, this study demonstrated that oral administration of osthole promotes healing progress by enhancing endochondral ossification in reparative phase with no significant influence on remodeling. We suggest that osthole is a potential osteoanabolic agent with which can be applied on osteoporotic fracture in long term with economical cost.
